# Retinoblastoma protein-initiated cellular growth arrest overcomes the ability of cotransfected wild-type p53 to induce apoptosis

**DOI:** 10.1054/bjoc.2000.1411

**Published:** 2000-10

**Authors:** H Shinohara, J Zhou, K Yoshikawa, S Yazumi, K Ko, Y Yamaoka, T Mizukami, T Yoshida, S Akinaga, T Tamaoki, H Motoda, W F Benedict, R Takahashi

**Affiliations:** Departments of Pathology and Tumour Biology, Gastroenterological Surgery, Graduate School of Medicine, Kyoto University, Yoshidakonoe-cho, Sakyo-ku, Kyoto, 606-8501, Japan; Department of Molecular Hematology and Therapy, The University of Texas MD Anderson Cancer Center, 1515 Holcombe Boulevard, Houston, Texas, 77030, USA; Pharmaceutical Research Institute, Kyowa Hakko Kogyo Co., Ltd., 1188 Shimotokari, Nagaizumi-cho, Sunto-gun, Shizuoka, 411-8731, Japan

**Keywords:** retinoblastoma gene, p53 gene, transfection, apoptosis, cell cycle

## Abstract

The retinoblastoma gene, RB, participates in the regulation of the G1/S-phase transition and in p53-mediated apoptosis. We have previously reported that stably transfected RB functions as a growth and tumour suppressor in HTB9 human bladder carcinoma cells, which carry a mutation of the p53 gene at codon 280 and lack RB expression. To elucidate the potential role of RB in the regulation of p53-mediated apoptosis, we transfected a wt p53 expression plasmid under the control of the human cytomegalovirus promoter into parental and RB-transfected HTB9 cells. The p53^+^/RB^–^ cells were susceptible to apoptosis under various experimental conditions: 1) incubation in serum-free culture for 72 h, 2) short-term (6 h) or long-term (48 h) exposure to etoposide, and 3) culturing in soft agar. In contrast, p53^+^/RB^+^ cells were significantly resistant to apoptosis under similar conditions and exhibited efficient growth arrest, as measured by laser scanning cytometry. Tumorigenicity in nude mice of parental HTB9 cells was lost by exogenous expression of wt p53. Likewise, none of mice injected subcutaneously with either p53^–^/RB^+^ or p53^+^/RB^+^ cells developed tumours, indicating that RB allows suppression of tumorigenesis, regardless of p53 status. These results suggest that the growth-inhibitory function of RB may overcome the ability of wt p53 to induce apoptosis. © 2000 Cancer Research Campaign


*British Journal of Cancer* (2000) **83**(8), 1039–1046
© 2000 Cancer Research Campaign
doi: 10.1054/ bjoc.2000.1411, available online at http://www.idealibrary.com on IDEAL®

### Retinoblastoma protein-initiated cellular growth arrest overcomes the ability of cotransfected wild-type p53 to induce apoptosis

**H Shinohara[1,2], J Zhou[3], K Yoshikawa[1,2], S Yazumi[1], K Ko[1], Y Yamaoka[2], T Mizukami[4], T Yoshida[4], S Akinaga[4], T Tamaoki[4], H Motoda[1], WF Benedict[3] and R Takahashi[1]**

Departments of [1]Pathology and Tumour Biology and [2]Gastroenterological Surgery, Graduate School of Medicine, Kyoto University, Yoshidakonoe-cho, Sakyo-ku, Kyoto 606-8501, Japan; [3]Department of Molecular Hematology and Therapy, The University of Texas MD Anderson Cancer Center, 1515 Holcombe Boulevard, Houston, Texas 77030, USA; [4]Pharmaceutical Research Institute, Kyowa Hakko Kogyo Co., Ltd., 1188 Shimotokari, Nagaizumi-cho, Sunto-gun, Shizuoka 411-8731, Japan.

**Summary** The retinoblastoma gene, RB, participates in the regulation of the G1/S-phase transition and in p53-mediated apoptosis. We have previously reported that stably transfected RB functions as a growth and tumour suppressor in HTB9 human bladder carcinoma cells, which carry a mutation of the p53 gene at codon 280 and lack RB expression. To elucidate the potential role of RB in the regulation of p53-mediated apoptosis, we transfected a wt p53 expression plasmid under the control of the human cytomegalovirus promoter into parental and RB-transfected HTB9 cells. The p53$^+$/RB$^-$ cells were susceptible to apoptosis under various experimental conditions: 1) incubation in serum-free culture for 72 h, 2) short-term (6 h) or long-term (48 h) exposure to etoposide, and 3) culturing in soft agar. In contrast, p53$^+$/RB$^+$ cells were significantly resistant to apoptosis under similar conditions and exhibited efficient growth arrest, as measured by laser scanning cytometry. Tumorigenicity in nude mice of parental HTB9 cells was lost by exogenous expression of wt p53. Likewise, none of mice injected subcutaneously with either p53$^-$/RB$^+$ or p53$^+$/RB$^+$ cells developed tumours, indicating that RB allows suppression of tumorigenesis, regardless of p53 status. These results suggest that the growth-inhibitory function of RB may overcome the ability of wt p53 to induce apoptosis. © 2000 Cancer Research Campaign

**Keywords**: retinoblastoma gene; p53 gene; transfection; apoptosis; cell cycle

The p53 tumour suppressor gene regulates a DNA damage-triggered G1 checkpoint (Hartwell and Kastan, 1994). p53-dependent growth arrest occurs upon transcriptional activation of p21, which in turn inhibits cyclin-dependent kinase-mediated phosphorylation of retinoblastoma protein (pRB) (Shaw et al, 1992; Clarke et al, 1993; Hartwell and Kastan, 1994). In contrast, loss of retinoblastoma gene (RB) function, both in cultured cells and in animals, results in inappropriate S-phase entry and apoptosis (Morgenbesser et al, 1994; Almasan et al, 1995; Macleod et al, 1996). RB, therefore, plays an important role in the regulation of p53-mediated apoptosis.

Induction of p53-mediated apoptosis is enhanced by: lack of expression of p21, an upstream regulator of RB (Waldman et al, 1996); viral E1A protein, which binds and inactivates pRB (White et al, 1991); and overexpression of E2F-1, a downstream protein whose action to drive the G1/S-phase transition is regulated by RB (Kowalik et al, 1995; Qin et al, 1994). Conversely, coexpression of functional RB with concomitant overexpression of wild-type (wt) p53 can overcome p53-dependent apoptosis in HeLa cells (Haupt et al, 1995). All of these observations suggest that p53 and RB play opposing roles in the control of apoptosis, that is, RB serves as a barrier to p53-mediated apoptosis by regulating the G1/S-phase transition. There is little direct evidence, however, to substantiate this hypothesis, especially in human cancer cells.

*Received 7 July 1999*
*Revised 28 June 2000*
*Accepted 28 June 2000*

*Correspondence to:* R Takahashi

We previously reported that the stably transfected RB gene functions as a growth suppressor in HTB9 human bladder carcinoma cells, which carry a mutation of the p53 gene at codon 280 and lack RB expression (Takahashi et al, 1991). In the present study, we employed one of these clones to obtain double transfectants that coexpressed RB and wt p53. This study addresses the potential role of RB in the regulation of p53-mediated apoptosis by using wt p53/RB variants derived from parental HTB9 cells. We show by cell cycle analyses and soft agar assays that the growth-arrest function of RB leads to suppression of wt p53-mediated apoptosis.

### MATERIALS AND METHODS

### Reagents

RPMI 1640 medium was purchased from GIBCO/BRL (Rockville, MD, USA). Fetal bovine serum (FBS) was purchased from Whittacker (Walkersville, MD, USA). $Ca^{2+}$- and $Mg^{2+}$-free phosphate-buffered saline (PBS), etoposide, propidium iodide (PI) and ribonuclease A (RNase A) were purchased from Sigma Chemical Co (St. Louis, MO, USA).

### Mice

Athymic 4-week-old Balb/c nude mice were purchased from Clea (Tokyo, Japan). The mice were maintained according to institutional guidelines in accordance with the National Institutes of Health (1996).

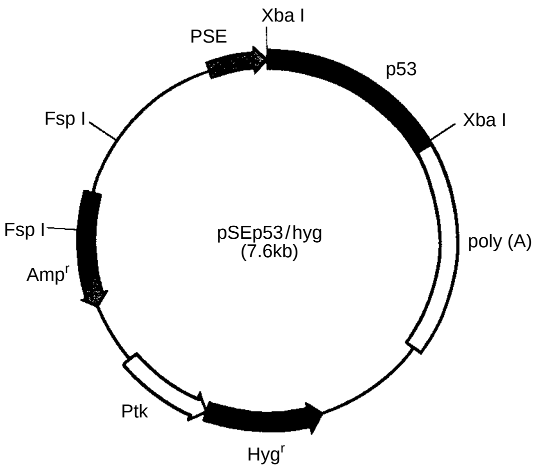

**Figure 1** Schematic presentation of wt p53 expression plasmid. p53 = wt p53-cDNA insert; Hyg$^r$ = hygromycin B-resistant gene; Ptk = phosphothymidine kinase promoter; Amp$^r$ = ampicillin-resistant gene; PSE = SV40 early promoter

### Cell lines

The parental HTB9 cells and the stably RB-transfected clone, H/RB (Takahashi et al, 1991) were cultured at 37°C in RPMI 1640/25 mM HEPES medium supplemented with 10% FBS and 1% penicillin-streptomycin in an atmosphere of 5% $CO_2$.

### Stable transfection of wt p53

We constructed a wt p53-expression plasmid, pSEp53/hyg (Figure 1), by inserting the isolated wt p53 cDNA and its entire coding region under control of the SV40 early promoter into expression vector pAGE208 (data not shown). H/RB cells were plated onto 100-mm culture dishes at a density of $5 \times 10^5$ per dish and transfected by using a mammalian transfection kit (Stratagene, La Jolla, CA, USA) with the plasmid digested with *Fsp* I. After 3 weeks, the hygromycin B-resistant clones were maintained separately in medium containing hygromycin B (200 μg ml$^{-1}$) and were propagated for further analysis. In another transfection experiment, the wt p53-expression plasmid pCDM8-p53/neo, in which expression of the p53 gene is under the control of the CMV promoter (Tamura et al, 1995), was digested with *Sca* I and transfected into HTB9 cells, yielding G418-resistant clones (200 μg ml$^{-1}$).

### Reverse transcriptase-initiated polymerase chain reaction (RT-PCR) and cDNA sequencing

Total cytoplasmic RNA was isolated according to the guanidium thiocyanate method (Chomczynski and Sacchi, 1987). For cDNA sequence analysis, cDNA was synthesized from 1 μg of total RNA by using a First Strand Synthesis Kit (Pharmacia, Piscataway, NJ, USA). Since parental HTB9 cells are known to have a missense mutation at codon 280, a region corresponding to exons 4 through 10 of the p53 gene was amplified from the cDNA by PCR using a set of primers, 5′-CTTCTGTCCCTTCCCAGAAAACC-3′ and 5′-CCTCATTCAGCTCTCGGA ACATCTCG-3′. cDNA sequencing was performed by using an *fmol* cycle sequencing kit (Promega, Madison, WI, USA) with a primer corresponding to the 3′-end of exon 7 (5′-XTAGTGTGGTGGTGCCCTATGAGCCG-3′, X: Biotin). The sequence ladder was detected by using a Sequencing High chemiluminescent detection kit (Toyobo, Osaka, Japan). Using primers, 5′-ATGATGTGTTCCATGTATGGC-3′ and 5′-AATATAGATGTTCCCTCCAGG-3′, we performed RT-PCR to amplify the RB gene from cDNA.

### Northern blot analysis

RNA blotting was performed as described by Thomas (1980). The membranes were then hybridized with a digoxigenin-labelled, 1.3-kb p53-RNA probe by using a DIG RNA-labeling kit (Boehringer Mannheim GmbH, Mannheim, Germany). Endogenous and exogenous p53-mRNAs were detected by using a DIG nucleic acid detection kit (Boehringer Mannheim GmbH).

### Immunocytochemistry

Cells were cultured at subconfluence in a Lab-Tek chamber slide (Nunc, Naperville, IL, USA). After being fixed with neutral-buffered formalin, the cells were permealized for 5 min in PBS containing 0.1% Triton X-100. The endogenous peroxidase activity was blocked by incubating the cells for 10 min in 3% $H_2O_2$. The cells were then incubated at room temperature for 2 h with the anti-RB antibody (MBL Inc, Nagoya, Japan) or with the anti-p16$^{INK4A}$ antibody (Santa Cruz, Santa Cruz, CA, USA) and for 30 min with biotinylated horse anti-mouse IgG. The slide was incubated for 30 min with the avidin–biotin peroxidase complex reagent (Vecstatin ABC kit, Vector Laboratories, Burlingame, CA, USA). Diaminobenzidine (0.05%) was used as the final chromogen, and haematoxylin was used as the nuclear counterstain.

### Immunoblotting

Cell extracts containing 100 μg protein were separated electrophoretically by using either 5% or 4–20% SDS-PAGE (Daiichi Pure Chemicals, Tokyo, Japan). The proteins were transferred onto a PVDF membrane (Immobilon-P; Millipore, Tokyo, Japan) for 2 h at 200 mA. The membrane was blocked with 5% nonfat dried milk in phosphate-buffered saline (PBS) containing 0.1% Tween-20. For detecting RB, the anti-RB monoclonal antibody (MBL, Nagoya, Japan) was used as a primary antibody. For p16, the anti-p16$^{INK4A}$ antibody (PharMingen, San Diego, CA, USA) was used as a primary antibody. The secondary antibody used for both RB and p16, was horseradish peroxidase-conjugated sheep anti-mouse IgG (H + L) (Amersham, Buckinghampshire, UK). The antibody complexes were detected by using an enhanced chemiluminescence system (Amersham).

### Cell-cycle analysis

Cells ($7 \times 10^4$) in complete medium containing 10% FBS were seeded on sterile glass slides. After 24 h of plating, the cells were cultured in 100-mm dishes in medium without 10% FBS or with 10% FBS as a control. Etoposide, an inhibitor of DNA topoisomerase II that ultimately causes apoptotic cell death by p53-dependent pathways (Clarke et al, 1993), was dissolved in dimethyl sulphoxide, added to the medium containing 10% FBS at a final concentration of either 6 or 30 μg ml$^{-1}$, and allowed to react for up to 48 h before fixation. After 96 h, the cells growing on the slides were fixed in 99% ethanol at 4°C overnight, stained with a PI

 *© 2000 Cancer Research Campaign*

(50 μg ml$^{-1}$) solution containing 100 μg ml$^{-1}$ of RNase A, and then incubated at 37°C for 30 min. Cells that had adhered to the slides in an area measuring 5 × 5 mm were scanned with an LSC101 laser scanning cytometer (Olympus Optics Co, Tokyo, Japan) nuclear debris and overlapping nuclei were gated out by statistical filters (Kamentsky and Kamentsky, 1991). The percentage of viable cells was calculated by using the following formula: viability (%) = A/B × 100, where A is the number of cells in treated cultures and B is the number of cells in the control cultures. The statistical significance of the differences in viability was determined by performing unpaired Student's *t*-test. DNA ploidy was determined by analysing DNA histograms that were generated by using computer software, as in flow cytometry, and the cells whose ploidy was abnormal were recalled to observe their nuclear morphology.

### Soft agar assay

Cells were seeded at a density of 1 × 10$^5$ per 60-mm dish in RPMI medium containing 0.33% agarose and 20% FBS. The medium was replenished every 7 days. The colonies (>50 cells each) were scored after 3 weeks, and the growth results were calculated as the average of three culture dishes per cell strain.

### Tumorigenesis

Cultures of parental and transfected tumour cells in their exponential growth phase were harvested by a brief exposure to a solution of 0.25% trypsin and 0.1% EDTA. The cell suspension was pipetted to produce a single-cell suspension, and then washed and resuspended in PBS. Cells (1 × 10$^7$ in 0.2 ml PBS) were injected subcutaneously into the right flank of a nude mouse. Tumour volume was measured every 1–2 weeks for 6 weeks. To re-establish a line from the tumours in culture the tumours were minced under sterile conditions and transferred to culture dishes in the presence of complete medium containing either G418 or hygromycin.

### DNA sequencing

Alterations with coding sequences of the p16 gene were tested by sequencing of the PCR amplified gene fragments by using a LI-COR Model 4000 DNA sequencer (Aloka, Tokyo, Japan) following the manufacturer's protocol. Each PCR product was cloned into the TA cloning vector and its sequence was analysed in each of three clones.

### RESULTS

### Characterization of transfected clones

Nineteen separate hygromycin B-resistant clones were obtained by transfecting the wt p53 gene into the H/RB cells that we had previously isolated by transfecting the RB gene into parental HTB9 cells (Takahashi et al, 1991). Transcripts from eight clones were sequenced to identify the exogenous wt-p53 allele after RT-PCR. Both wt and mutant p53 sequences that corresponded to codon 280 were identified in six clones, namely H/RB/p53-1, -2, -4, -5, -7, and -8. Representative results for HTB9 and one of these clones, H/RB/p53-1, are shown in Figure 2A. The RNAs of these clones

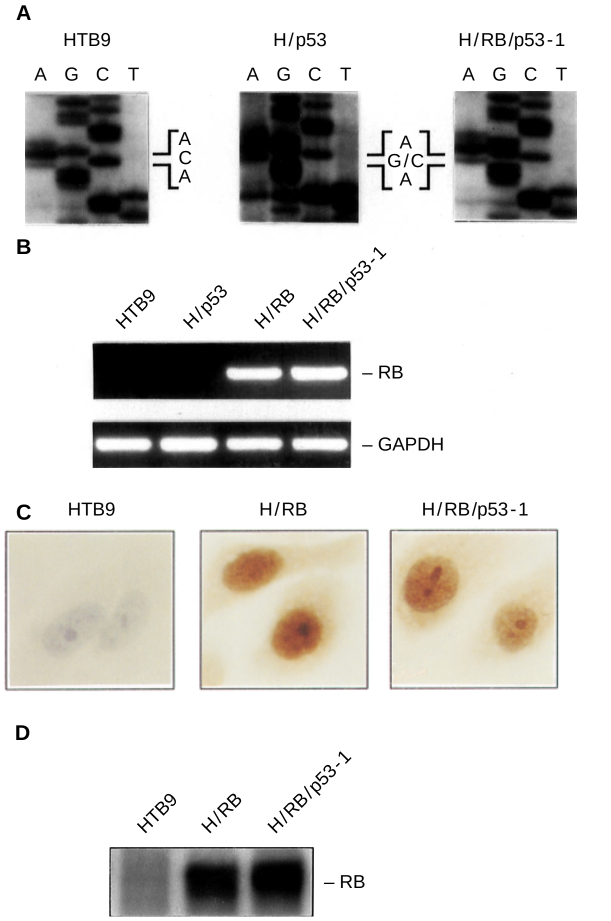

**Figure 2** Identification of exogenous wt p53 and RB gene expression (**A**) RT-PCR sequencing analysis of parental HTB9, H/p53, and H/RB/p53-1 cells. The sequence corresponding to codon 280 is indicated. Both the wt and the mutant p53 sequences were identified in H/p53 and in H/RB/p53-1 cells. (**B**) RT-PCR for detecting RB gene expression (upper panel) and glyceraldehyde phosphate dehydrogenase (GAPDH) gene expression (lower panel). (**C**) Demonstration of nuclear RB expression by immunocytochemistry using anti-RB antibody (magnification × 400). (**D**) Demonstration of RB expression by Western blotting were also analysed by Northern blotting to identify exogenous p53 mRNA. The exogenous wt p53-mRNA (2.1-kb), which could be distinguished from the mutant p53-mRNA (2.8 kb) by its size, was also detected by Northern blotting except in the H/RB/p53-3 and -6 clones (data not shown). Also confirmed was the fact that, like H/RB cells, all of the eight clones were resistant to G418 at a dose of 400 μg ml$^{-1}$. RT-PCR revealed that the expression levels of the exogenous RB gene in wt p53 transfectants were persistent (Figure 2B), although the levels were relatively lower in H/RB/p53-2 and -8 cells by Northern blotting (data not shown). Furthermore, as measured by immunostaining with monoclonal anti-RB antibody, nuclear staining of pRB in H/RB/p53-1, -2, -5, and -8 clones was similar to that in the H/RB cells. The staining pattern of H/RB/p53-1 and H/RB cells is represented in Figure 2C. The expression levels of exogenous RB protein were mostly equivalent among the RB-transfected clones analysed by immunoblotting using anti-RB monoclonal antibody. The representatives are shown in Figure 2D. Therefore, for subsequent studies, we selected H/RB/p53-1 as a representative cotransfectant with RB

© 2000 Cancer Research Campaign

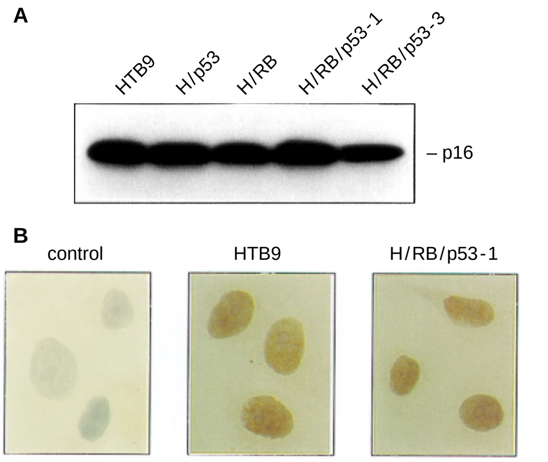

**Figure 3** Expression of the p16[INK4A] protein before and after transfection. (**A**) Western blotting of parental HTB9, H/p53, H/RB, H/RB/p53-1 and H/RB/p53-3 cells. (**B**) Immunocytochemical staining. Control = without primary antibody; HTB9 and H/RB/p53-1 = with the p16[INK4A] antibody and wt p53, and H/RB/p53-3 was used as a negative control for wt p53 expression.

We also transfected the wt p53 expression plasmid into parental HTB9 cells to obtain $p53^+/RB^-$ clones. By using RT-PCR and cDNA sequencing, we identified exogenous wt p53 in three G418-resistant clones. Representative data for one of these clones, H/p53, are shown in Figure 2A.

The status of the p16 gene, which is one of the frequently mutated genes in the RB pathway, was also assessed by Western blotting and immunocytochemical staining (Figure 3). Western blotting showed that the expression level of the p16 protein was maintained in all the transfected clones. These data were also confirmed at the single-cell level by immunocytochemical staining using the anti-p16[INK4A] antibody. Furthermore, sequence analysis of the genomic p16 locus was also performed, and revealed no mutation in exons 1, 2 and 3 of the p16 gene (data not shown).

### RB prevents cells from losing viability when deprived of serum

We examined whether cell viability was associated with exogenous expression of the wt p53 and/or RB genes by using laser scanning cytometry (LSC). In control cultures that were incubated for 96 h with normal medium containing 10% FBS, the number of viable cells transfected with the wt p53 and/or RB genes was significantly lower than that of parental cells ($P < 0.01$); the mean numbers of viable HTB9, H/p53, H/RB, H/RB/p53-1, and H/RB/p53-3 cells were calculated to be 15187 ± 426, 9271 ± 713, 7860 ± 922, 8044 ± 323, and 7166 ± 197, respectively. Table 1 shows the viability of these cells when they were incubated with FBS-free medium for 72 h before fixation. The viability of the H/p53 cells decreased significantly, as compared to that of HTB9 cells. In sharp contrast, the H/RB, H/RB/p53-1, and H/RB/p53-3 cells were much more resistant to the effects of serum deprivation.

### RB suppresses S-phase entry and apoptosis in cells exposed short-term to etoposide

We induced apoptosis by treating the cells with etoposide, a DNA-strand breakage agent (Table 1, Figure 4). There were no significant differences in the viability of the cells that were treated with low-dose (6 μg $ml^{-1}$) etoposide for 6 h before fixation. LSC revealed that, in all cell groups, more than 40% of the cells reached S-phase, whereas cells in the G1-phase were much fewer when compared to the controls. Notably, the cell fraction in early S-phase was observed in parental HTB9 cells and in the wt p53-expressive clones H/p53 and H/RB/p53-1 but not in the purely pRB-positive clones H/RB and H/RB/p53-3. When treated with high-dose (30 μg $ml^{-1}$) etoposide, the viability of the H/p53 cells significantly decreased, as compared to that of the HTB9 cells. The H/p53 cells had a characteristic DNA histogram, showing cells in the sub-G1-phase and in a higher DNA ploidy cycle (i.e. additional-S-phase), which is consistent with the apoptotic process reported previously (Waldman et al, 1996). In contrast, the pRB-expressive cells, including the wt p53-positive H/RB/p53-1 cells, were much more resistant to the etopside treatment, and the cell population in the sub-G1- or additional-S-phase was insignificant (Figure 4). Exogenous expression of RB apparently protected human bladder carcinoma cells from p53-dependent apoptosis after short-term (6 h) exposure to etoposide.

### RB induces growth arrest at the G2/M-phase in cells exposed long-term to etoposide

We examined the effects of long-term exposure of etoposide on apoptotic cell death (Table 1, Figure 5). The viability of the H/p53 cells significantly decreased when the cells were treated for 48 h with 6 μg $ml^{-1}$ etoposide, as compared to that of the HTB9 cells.

**Table 1** Viability of human bladder carcinoma cells deprived of serum or treated with etoposide

| Cell strain | Expression status p53 | RB | Viability (%)[a] FBS free | Etoposide 6 μg $ml^{-1}$ (6 h) | 30 μg $ml^{-1}$ (6 h) | 6 μg $ml^{-1}$ (48 h) |
|---|---|---|---|---|---|---|
| HTB9 | mt | – | 25.9 ± 1.6 | 81.2 ± 1.5 | 61.3 ± 2.3 | 8.5 ± 7.4 |
| H/p53 | wt/mt | – | 16.3 ± 0.7[c] | 82.0 ± 2.6 | 36.9 ± 3.9[c] | 2.4 ± 0.3[c] |
| H/RB | mt | + | 60.3 ± 3.2[c] | 85.0 ± 2.9 | 87.0 ± 2.9[c] | 16.3 ± 14.1[b] |
| H/RB/p53-1 | wt/mt | + | 61.9 ± 2.0[c,d] | 84.4 ± 9.6 | 81.5 ± 1.3[c,d] | 13.7 ± 11.9[b,d] |
| H/RB/p53-3 | mt | + | 56.6 ± 5.4[c] | 89.1 ± 2.6 | 85.9 ± 3.0[c] | 16.9 ± 14.6[b] |

[a] The percentage of viable cells was calculated by using the following formula: viability (%) = A/B × 100, where A is the number of cells in the treated cultures and B is the number of cells in the control cultures. Results are presented as means ± SD of five different areas of the slides; [b] $P < 0.01$, as compared to HTB9 cells; [c] $P < 0.001$, as compared to HTB9 cells; [d] $P < 0.0001$, as compared to H/p53 cells.
FBS = fetal bovine serum.

 *© 2000 Cancer Research Campaign*

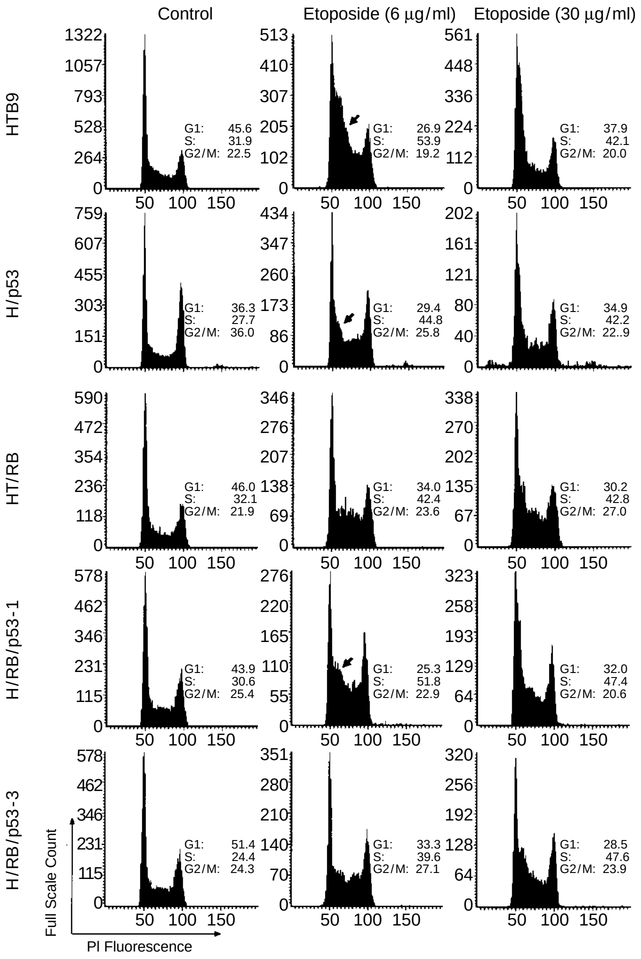

**Figure 4** Laser scanning cytometric analysis of human bladder carcinoma cells treated with or without etoposide for 6 h before fixation. The histograms represent DNA content and indicate cells in the G1-, S-, or G2/M-phase of the cell cycle as determined by staining with propidium iodide. Note the cell fraction in early S-phase (arrows) observed in parental HTB-9, H/p53, and H/RB/p53-1 cells treated with 6 μg ml$^{-1}$ of etoposide

Many of H/p53 cells were found to be dead or dying by phase-contrast microscopy (Figure 5A). In contrast, the pRB-expressive cells, including the wt p53-positive H/RB/p53-1 cells, were much more resistant to this treatment. LSC revealed that the pRB-expressive H/RB and H/RB/p53-1 cells were arrested at the G2/M-phase, regardless of p53 status. The peak of the DNA histogram of the pRB-nonexpressive parental HTB-9 and H/p53 cells was shifted to the left, however, and the cells had accumulated in an S-like state. Furthermore, a large accumulation of H/p53 cells had entered the additional-S-phase (Figure 5B). Representative H/p53 cells with sub-G1, S-like, and additional-S DNA content were recalled and photographed (Figure 5C). Fragmented and highly condensed chromatin was seen in sub-G1 cells, whereas cells in the additional-S-phase had large nuclei. In contrast to H/RB/p53-1 cells in the G2-phase, cells defined as being in an S-like state exhibited very little residual DNA staining, probably because of the loss of degraded DNA from cells in the advanced stages of apoptosis.

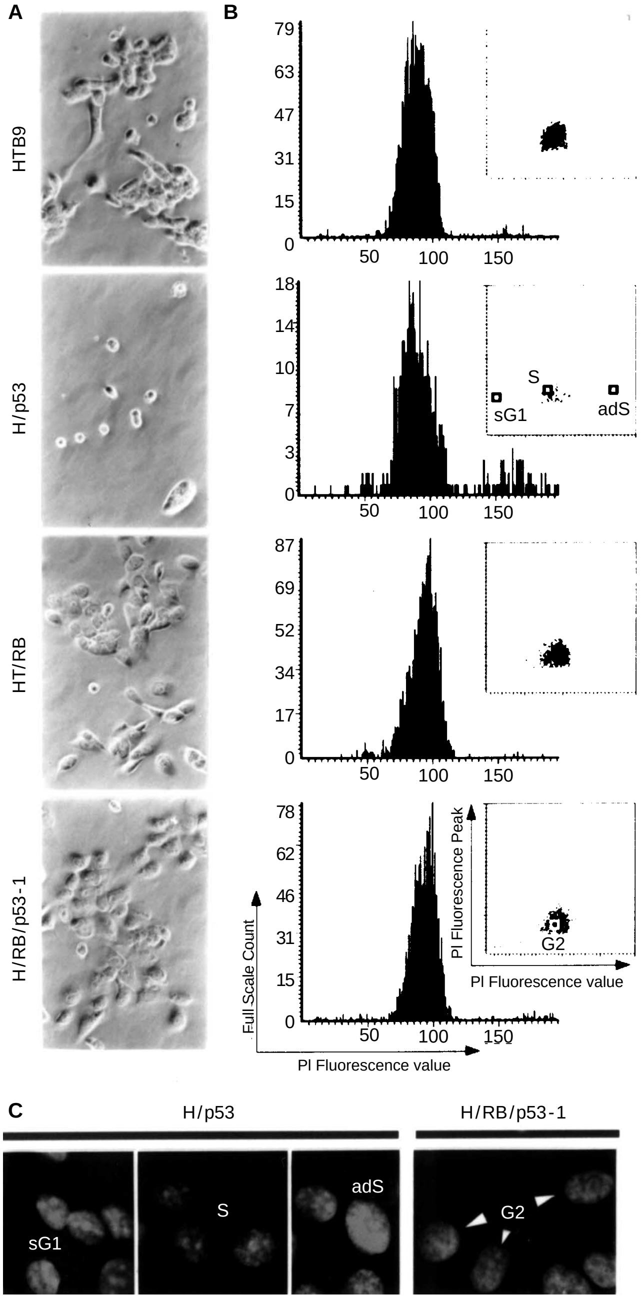

**Figure 5** Apoptosis in human bladder carcinoma cells treated with 6 μg ml$^{-1}$ of etoposide for 48 h before fixation. (**A**) Cell viability as measured by phase-contrast microscopy (magnification × 400). (**B**) Histograms representing DNA content as measured by LSC. Most of the H/RB and H/RB/p53-3 cells were arrested at the G2/M-phase, whereas the peak of the histogram was shifted to the left in parental HTB-9 and H/p53 cells. Small squares (□) indicate cells recalled for the photographs shown in **C**. (**C**) Representative H/p53 cells that had sub-G1 (sG1)-, S-like (S)-, and additional-S (adS)-phase DNA content, and H/RB/p53-1 cells that had G2-phase DNA content. Cells were recalled from the DNA histogram, and photographed by a 3CCD camera attached to the microscope of the cytometer (magnification × 400)

### RB increases colony formation in soft agar

We examined the ability of human bladder carcinoma cells to form colonies in soft agar to determine the effects of anchorage dependency on induction of apoptosis. As shown in Table 2, the H/p53

© 2000 Cancer Research Campaign

**Table 2** Growth in soft agar and tumorigenicity of human bladder carcinoma cells transfected with the wt p53 or RB gene or both

| Cell strain | Expression status | | Colonies in soft agar[a] | Mice with tumours/ mice injected[b] | Average tumour size[c] ($mm^3$) |
|---|---|---|---|---|---|
| | p53 | RB | | | |
| HTB9 | mt | – | 299 | 3/3 | 74.2 |
| H/p53 | wt/mt | – | 6 | 0/3 | 0 |
| H/RB | mt | + | 702 | 0/4 | 0 |
| H/RB/p53-1 | wt/mt | + | 562 | 0/5 | 0 |
| H/RB/p53-3 | mt | + | 788 | 5/5 | 33.6 |

[a] Cells were seeded at a density of $1 \times 10^5$ per 60-mm dish in RPMI medium containing 0.33% agarose and 20% fetal bovine serum. The values were calculated as the average of three culture dishes per cell strain; [b] Nude mice were injected with $1 \times 10^7$ cells into the subcutis of the right flank; [c] Cubic volumes are based on three-dimensional measurements of the tumours.

cells almost completely lost the ability to from colonies in soft agar. In contrast, the number of colonies formed by the pRB-expressive H/RB, H/RB/p53-1, and H/RB/p53-3 clones, were significantly greater than those formed by parental HTB9 cells.

### RB inhibits tumorigenesis in nude mice

In the final set of experiments, we investigated the effect of stable RB or p53 transfection, or both, on the tumorigenicity of human bladder carcinoma cells (Table 2). All of the mice injected with parental HTB9 cells produced tumours that grew progressively. In contrast, none of mice injected with H/p53 cells produced tumours. As we reported previously (Takahashi et al, 1991), the H/RB cells did not exhibit tumorigenicity in nude mice, nor did the H/RB/p53-1 cells coexpressing wt p53 and RB. These results indicated that RB allows suppression of tumorigenesis, regardless of p53 status. Unexpectedly, all five animals injected with H/RB/p53-3 cells formed tumours. Histopathological analysis disclosed that tumours derived from the H/RB/p53-3 cells retained the malignant characteristics of the parental cells with moderate differentiation (data not shown). Cells from each of the tumours that were re-established in cultures were found to have significantly lower expression of RB mRNA, as compared with originally injected cells, in addition to the negative expression of wt p53 mRNA (data not shown).

### DISCUSSION

Despite the strong inhibitory effects of tumour suppressor genes on cell growth, the isolation of wt p53- or RB-transfected clones by using a plasmid has occasionally been successful (Takahashi et al, 1991; Tamura et al, 1995). In this study, we successfully isolated, for the first time, clones that stably express both wt p53 and RB under the control of noninducible promoters in human carcinoma cells that carry a mutation of the p53 gene and lack RB expression. Our data demonstrate that exogenous coexpression of pRB can suppress wt p53-mediated apoptosis in cells deprived of serum or treated with etoposide. These findings are also supported by soft agar assays that demonstrated that exogenous expression of pRB is sufficient to promote colony formation, which overcomes the inability of anchorage-independent cellular growth mediated by wt p53.

RB is an important cell-cycle regulator, acting to control the G1/S-phase transition (Bartek et al, 1997). Absence of RB function is known to allow synthesis of gene products required for S-phase entry (Almasan et al, 1995) and to activate p53-dependent apoptosis (Morgenbesser et al, 1994; Almasan et al, 1995; Macleod et al, 1996). At the molecular level, phosphorylation of pRB results in the activation of E2F transcription factors, which in turn activate the transcription of several S-phase genes including cyclin E, cyclin D, and c-myc (Yamasaki, 1998). Interestingly, recent studies have demonstrated paradoxical roles for E2F-1 in the induction of p53-dependent apoptosis (Qin et al, 1994; Kowalik et al, 1995; Pan et al, 1998; Tsai et al, 1998). Furthermore, overexpression of E2F-1 leads to increased levels of p53, and the apoptotic effect of E2F-1 can be overcome through coexpression of MDM2, a ubiquitin-like inhibitor of p53 (Kowalik et al, 1998). The sequence of evidence indicates that p53-dependent apoptosis is accelerated by S-phase entry, which is triggered by activation of E2F-1 resulting from loss of RB function. Our data also show that exogenous expression of pRB is sufficient to suppress apoptosis induced by serum deprivation or exposure to etoposide in wt p53-expressive cells. Moreover, LSC followed by treatment with low-dose etoposide demonstrated that the cell fraction in early S-phase appears in the wt p53-positive clones, regardless of RB status, but not in the purely pRB-positive clones. These results suggest that balanced levels of a series of molecules (e.g. E2F-1), when regulated by both p53 and RB, may control successful entry into S-phase.

The E2F-1 target genes that act upstream of p53 have yet to be identified. One candidate is $p19^{ARF}$, which can be induced by E2F-1 (DeGregori et al, 1997) and can cause p53 stabilization by inhibiting MDM2 (Pomerantz et al, 1998). We are currently investigating whether the levels of $p19^{ARF}$ are affected in our transfectants.

Agents, such as etoposide, that characteristically cause DNA strand breakage induce cellular growth arrest at two well-defined checkpoints, the G1/S and G2/M boundaries (Del Bino and Darzynkiewicz, 1991; Wyllie et al, 1996), and prevent cells from initiating either DNA synthesis or mitosis in the presence of strand breaks. Our LSC data that showed how cells treated with etoposide for 48 h accumulated at the G2/M-phase before apoptotic cell death (Figure 4), therefore, are reasonable and consistent with the results of previous studies involving HCT116 human colorectal cancer cells (Waldman et al, 1996) and human lymphocytic leukaemic MOLT-4 cells (Del Bino and Darzynkiewicz, 1991). Accumulation of p53 in cells arrested at the G2/M checkpoint also may be sufficient to accelerate apoptosis (Haapajärvi et al, 1995). Our results show that H/p53 cells, but not H/RB/p53-1 cells, are more susceptible to apoptosis and exhibit a characteristic DNA histogram, some H/p53 cells were arrested in the sub-G1- and

 © 2000 Cancer Research Campaign ectopic S-phase (S-like and additional-S). The specific role of RB in the suppression of apoptosis through G2/M arrest is under investigation.

Recent work on the type I insulin-like growth factor receptor, which allows cancer cells to maintain unregulated growth and to resist apoptosis, has demonstrated that tumorigenesis in nude mice is strictly dependent on the fraction of cells that escape apoptosis (Resnicoff et al, 1995; Singleton et al, 1996). Our results, however, indicate that exogenous RB expression is involved in the reduction of tumorigenesis, suggesting that the growth-arrest function of RB may be employed, not only to prevent apoptosis, but also to suppress tumour growth. Since pRB is known to repress E2F transcriptional activity and since overexpression of E2F is sufficient for cell-cycle progression, pRB apparently suppresses tumour growth in part by repressing E2F-mediated transcription (Yamasaki et al, 1998).

Quantitation of apoptosis is a critical part of the assay, and may not be easily achieved especially for attached cells. However, even by using a laser scanning cytometer, quantitation of sub-G1 cells does not represent total apoptotic cells, due to detachment of apoptotic cells from the bottom of the culture flasks. Although we confirmed DNA ladder formation in detached cells from the supernatant, it was not quantitive. One of the reasons why we used laser scanning cytometry is its capability to assess cell viability and morphology, which should also provide information on the rate of cell proliferation in certain populations of the cell cycle. This allowed us to normalize the number of cells. For example, the difference in the number of expected dividing cells between etoposide-treated and non-treated groups for 6 h was less than 5%. Therefore, the difference in the number of remaining cells can mostly be considered due to apoptosis.

Some investigators have proposed that RB can serve as a molecular switch to determine whether the activation of wt p53 will lead to growth arrest or to apoptosis (Qin et al, 1994; Haupt et al, 1995; Waldman et al, 1996). Our present findings clearly demonstrate that induction of functional RB results in suppression of wt p53-mediated apoptosis in human bladder cancer cells. Our results also suggest that apoptosis possibly occurred after the cells continued cycling and entered the S-phase and that RB possibly had an anti-apoptotic function by providing the genetically damaged cells with sufficient growth arrest. These possibilities may explain our in vivo data that showed how exogenous RB eradicates tumorigenicity despite inhibition of apoptosis. We believe our transfectants from the human bladder carcinoma cell line HTB9 will be a valuable model for directly analysing the mechanism of apoptosis and for testing the differential sensitivity of tumour cells, with or without wt p53 or RB expression, to various anticancer agents.

### ACKNOWLEDGEMENTS

This work was partly supported by a Grant-in-Aid from the Ministry of Education, Science, and Culture of Japan (09254230) and a grant from Kyowa Hakko Kogyo Co Ltd to RT, by a Research Fellowship of the Japan Society for the Promotion of Science (2655) to HS, and by grants from the National Institutes of Health (EY06196) and the Retina Research Foundation to WFB. We thank Pamela Paradis Tice, ELS, for editing the manuscript.

### REFERENCES

Almasan A, Yin Y, Kelly RE, Lee EY-HP, Bradley A, Li W, Bertino JR and Wahl GM (1995) Deficiency of retinoblastoma protein leads to inappropriate S-phase entry, activation of E2F-responsive genes, and apoptosis. *Proc Natl Acad Sci USA* **92**: 5436–5440

Bartek J, Bartkova J and Lukas J (1997) The retinoblastoma protein pathway in cell cycle control and cancer. *Exp Cell Res* **237**: 1–6

Chomczynski P and Sacchi N (1987) Single-step method of RNA isolation by acid guanidium thiocyanate-phenol-chloroform extraction. *Anal Biochem* **162**: 156–159

Clarke AR, Purdie CA, Harrison DJ, Morris RG, Bird CC, Hooper ML and Wyllie AH (1993) Thymocyte apoptosis induced by p53-dependent and independent pathways. *Nature* **362**: 849–852

DeGregori J, Leone G, Miron A, Jakoi L and Nevins JR (1997) Distinct roles for E2F proteins in cell growth control and apoptosis. *Proc Natl Acad Sci USA* **94**: 7245–7250

Del Bino G and Darzynkiewicz Z (1991) Camptothecin, tenioside, or 4′-(9-acridinylamino)-3-methanesulfon-m-anisidide, but not mitoxantrone or doxorubicin, induces degradation of nuclear DNA in the S phase of HL-60 cells. *Cancer Res* **51**: 1165–1169

Haapajärvi T, Kivinen L, Pitkänen K and Laiho M (1995) Cell cycle-dependent effects of UV-radiation on p53 expression and retinoblastoma protein phosphorylation. *Oncogene* **11**: 151–159

Hartwell LH and Kastan MB (1994) Cell cycle control and cancer. *Science* **266**: 1821–1828

Haupt Y, Rowan S and Oren M (1995) p53-mediated apoptosis in HeLa cells can be overcome by excess pRB. *Oncogene* **10**: 1563–1571

Kamentsky LA and Kamentsky LD (1991) Microscope-based multiparameter laser scanning cytometer yielding data comparable to flow cytometry data. *Cytometry* **12**: 381–387

Kowalik TF, DeGregori J, Schwarz JK and Nevins JR (1995) E2F-1 overexpression in quiescent fibroblasts leads to induction of cellular DNA synthesis and apoptosis. *J Virol* **69**: 2491–2500

Kowalik TF, DeGregori J, Leone G, Jakoi L and Nevins JR (1998) E2F-1-specific induction of apoptosis and p53 accumulation, which is blocked by MDM2. *Cell Growth Differ* **9**: 113–118

Macleod K, Hu Y and Jacks T (1996) Loss of Rb activates both p53-dependent and independent cell death pathways in the developing mouse nervous system. *EMBO J* **15**: 6178–6188

Morgenbesser SD, Williams BO, Jacks T and DePinho RA (1994) p53-dependent apoptosis produced by Rb-deficiency in the developing mouse lens. *Nature* **371**: 72–74

National Institutes of Health (1996) *Guide for the Care and Use of Laboratory Animals*. http://grants.nih.gov/grants/policy

Pan H, Yin C, Dyson NJ, Harlow E, Yamasaki L and Van Dyke T (1998) Key roles for E2F1 in signaling p53-dependent apoptosis and in cell division within developing tumours. *Mol Cell* **2**: 283–292

Pomerantz J, Schreiber-Agus N, Liegeois NJ, Silverman A, Alland L, Chin L, Potes J, Chen K, Orlow I, Lee HW, Cordon-Cardo C and DePinho RA (1998) The Ink4a tumour suppressor gene product, p19Arf, interacts with MDM2 and neutralizes MDM2's inhibition of p53. *Cell* **92**: 713–723

Qin X-Q, Livingston DM, Kaelin WG and Adams PD (1994) Deregulated transcription factor E2F-1 expression leads to S-phase entry and p53-mediated apoptosis. *Proc Natl Acad Sci USA* **91**: 10918–10922

Resnicoff M, Burgand JL, Rotman HL, Abraham D and Baserga R (1995) Correlation between apoptosis, tumorigenesis, and levels of insulin-like growth factor I receptors. *Cancer Res* **55**: 3739–3741

Shaw P, Bovey R, Tardy S, Sahli R, Sordat B and Costa J (1992) Induction of apoptosis by wild-type p53 in a human colon tumour-derived cell line. *Proc Natl Acad Sci USA* **89**: 4495–4499

Singleton JR, Randolph AE and Feldman EL (1996) Insulin-like growth factor I receptor prevents apoptosis and enhances neuroblastoma tumourigenesis. *Cancer Res* **55**: 3739–3741

Takahashi R, Hashimoto T, Xu H-J, Hu S-X, Matsui T, Miki T, Bigo-Marshall H, Aaronson SA and Benedict WF (1991) The retinoblastoma gene functions as a growth and tumour suppressor in human bladder carcinoma cells. *Proc Natl Acad Sci USA* **88**: 5257–5261

Tamura T, Aoyama N, Saya H, Haga H, Futami S, Miyamoto M, Koh T, Ariyasu T, Tachi M, Kasuga M and Takahashi R (1995) Induction of Fas-mediated apoptosis in p53-transfected human colon carcinoma cells. *Oncogene* **11**: 1939–1946

Thomas PS (1980) Hybridization of denatured RNA and small DNA fragments transferred to nitrocellulose. *Proc Natl Acad Sci USA* **77**: 5201–5205

© 2000 Cancer Research Campaign 

Tsai KY, Hu Y, Macleod KF, Crowley D, Yamasaki L and Jacks T (1998) Mutation of E2F-1 suppresses apoptosis and inappropriate S phase entry and extends survival of Rb-deficient mouse embryos. *Mol Cell* **2**: 293–304

Waldman T, Lengauer C, Kinzler KW and Vogelstein B (1996) Uncoupling of S phase and mitosis induced by anticancer agents in cells lacking p21. *Nature* **381**: 713–716

White E, Cipriani R, Sabbatini P and Denton A (1991) Adenovirus E1B 19-kilodalton protein overcomes the cytotoxicity of E1A proteins. *J Virol* **65**: 2968–2978

Wyllie FS, Haughton MF, Bond JA, Rowson JM, Jones CJ and Wynford-Thomas D (1996) S phase cell-cycle arrest following DNA damage is independent of the p53/p21WAF1 signalling pathway. *Oncogene* **12**: 1077–1082

Yamasaki L (1998) Growth regulation by the E2F and DP transcription factor families. In *Cell Cycle Control*, Pagano M (ed) pp 199–227. Springer–Verlag: Berlin

Yamasaki L, Bronson R, Williams BO, Dyson NJ, Harlow E and Jacks T (1998) Loss of E2F-1 reduces tumorigenesis and extends the lifespan of Rb1(+/–) mice. *Nature Genet* **18**: 360–364

*© 2000 Cancer Research Campaign*
